# Books

**Published:** 1883-05

**Authors:** 


### Books.

---

THE PATHOLOGY AND TREATMENT OF DISEASES OF THE OVARIES, (being the Hasting's Essay for 1883). By Lawson Tait, F.R.C.S., Edinburg and England, Surgeon to the Birmingham Hospital for Women and Consulting Surgeon for Diseases of Women to the West Bromwich Hospital, etc., etc. Pp. 351. New York: Wm. Wood & Co. 1883.

This is the fourth edition of the work, re-written and greatly enlarged. It is dedicated to Dr. Marion Sims, and contains, in the new and interesting field of abdominal surgery much that must greatly interest the profession.

In his preface to this addition, the author says: "I have seen far more reason for extending what I had to say; for the marvelous success which now attends the efforts of those who practice abdominal surgery has fallen largely to my lot, and therefore records of things accomplished, and opinions expressed upon them, will be found in these pages, which eight years ago, were certainly beyond the limits of acceptance."

On page 326, under the head of Recent Extensions of Abdominal and Pelvic Surgery, he again says: "The same idea, concerning the removal of small ovaries, had struck the minds of two other surgeons about the same time, as it occurred to me, for up to February 27, 1872, five days before my second case, Prof. Hegar, of Freiburg, removed both ovaries for neuralgia with a fatal result, and on the 17th August, of the same year, Dr. Battey, of Rome, Ga., successfully operated upon a patient suffering from serious and complicated symptoms. Dr. Battey was the first to publish his cases and a defense of his proceedings (*Atlanta Medical Journal*, September 1872), whilst I contented myself with discussing the principle only in my Hasting's Essay on "Diseases of the Ovary," (1873).

---

Students Guide to Disease of the Eye. By Edward Nettleship, F. R.C.S., Ophthalmic Surgeon to St. Thomas' Hospital, and to the Hospital for Sick Children, Great Ormond St. Second American from the Second Revised and Enlarged English Edition: With a Chapter on Examination for Color Perception. By Wm. Thomson, M.D., Professsor of Ophthalmology in the Jefferson Medical College. Pp. 416. Philadelphia: Henry C. Lea's Son & Co. 1883.

The class of persons for whom this manual has been prepared will appreciate the general carefulness and accuracy in so small a volume with which its distinguished author has done his work.

The American publisher, in his preface says: "In presenting to the medical profession the second American edition of Dr. Nettleship's Guide to Diseases of the Eye, the publiseers desire to state that no pains have been spared to place it in every particular upon a level with the latest developments of the specialty of which it treats."

A very important addition to this edition of the work, is the chapter by Dr. Wm. Thomas, on examination for color perception. This is a practical examination of railway employes as to their fitness for the very responsible position they hold requiring rapid and accurate discrimination between colored signals.

Heretofore there had been no means devised by which this could be done except by experts. Now by a very simple arrangement, herein detailed, any agent of a company can ascertain all the points desirable in such a test. We heartily commend this manual to students and railroad officials.

---

THERAPUUTIC HAND-BOOK OF THE UNITED STATES PHARMACOPIA. BEING A CONDENSED STATEMENT OF THE PHYSIOLOGICAL AND TOXIC ACTION, MEDICINAL VALUE, METHODS OF ADMINISTRATION AND DOSES OF THE DRUGS AND PREPARATIONS IN THE LATEST EDITION OF THE U. S. PHARMACOPIA. By Robert T. Edes, A.B., M.D. (Harvard) Fellow of the Massachusetts Medical Society, Etc. Pp. 397, New York: Wm. Wood & Co.

The aim of the author of this hand-brook of the pharmacopia, may be seen, at a glance, from his preface. "A National Pharmacopia should consist of a catalogue of all the drugs and preparations of acknowledged necessity and approved usefulness likely to be needed by the medical practitioner of any portion of the country. Such a catalogue must include as its principal and largest portion, the drugs employed over the whole civilized world, together with many others indigenous or easily obtained in the country for which it is intended."

This being true, it is obvious that for each individual practitioner, a small part only of the pharmacopia can ever be practically useful. To this part in each case, for the physicians of the United States is the object of the manual before us.

The therapeutical or physiological effects of each drug are given with the adult dose in both systems of weights.

To the work is appended a valuable classified index of drugs, together with a catalogue, for convenient reference, of poisons and their antidotes from the most recent and reliable sources of information.

---

A Manual of Auscultation and Percussion; Embracing the Physical Diagnosis of Diseases of the Lungs and Heart, and of Thoracic Aneurism. By Austin Flint, M. D. Third Edition Revised. Pp. 242. Philadelphia: Henry C. Lea's Son & Co. 1883.

The works of Dr. Flint speak for themselves. The speedy exhaustion of their successive editions, as of the second edition of this, is the best evidence of their value. We commend this little manual to students and physicians as the best of its kind now before the profession.

---

Transactions of the Medical Association of Georgia. Thirty-third Annual Session. 1883. Pp. 221. Augusta, Ga.

This meeting took place in the city of Atlanta, April 19th, and in the absence of the President, was called to order by Dr. James B. Baird, of Atlanta.

Dr. J. Thad. Johnson, of Atlanta, in the absence of the Sceretary, was appointed Secretary *pro tem.*

Dr. J. F. Alexander, of Atlanta, then delivered the address of welcome. Dr. Eugene Foster, of Augusta, responded in behalf of the Association. Dr. Baird then introduced the President elect, Dr. Wm. F. Holt, of Macon, and retired from the chair.

We regret the lack of space which forbids our giving such a review of the proceedings of this meeting as their value merits.

The report of the Secretary, Dr. A. Sibley Campbell, of Augusta, embraced in the volume of transactions just out, is minute, full and abounding in important suggestions to the members of the Association.

Judging from this report, we are of the opinion that the office of Secretary of the Medical Association of Georgia is not a sinecure, and its couch rather scarce of both roses and down. The volume of transactions is gotten up in a style that does infinite credit to the taste, judgment and energy of Dr. Campbell.

But in order to accomplish the work, he has obviously made great personal sacrifices and experienced sore trials. This part of his report, if written in Miltonian measure, would read like an epic. Whose fault is this? Either the members of the body must be more prompt in the payment of their dues, or some man of means among us must prepare for a comfortable death by endowing the Association.

---

MANUAL OF GYNÆCOLOGY. By D. Berry Hart, M.D., F.R.C.P.E. Lecturer on Midwifery and Diseases of Women, School of Medicine, Edinburg, etc., etc., and A. A. Bosbour, M.A., B.Sc., M.B., Assistant to the Professors of Midwifery of Edinburg, etc., etc. Vol, II, with one lithograph and two hundred and ten wood cuts. New York: Wm. Wood & Co. 1883.

It is hardly fair to pronounce judgment on an author's work from an odd volume. The one before us, the February, 1883, No. of Wood's Library, is issued in the usual handsome style of that house, and if in keeping with what is said by others of the first volume, the entire work is a valuable contribution to the Gynæcological branch of the profession.

---

THE DISEASES OF WOMEN—A MANUAL FOR PHYSICIANS AND STUDENTS. By Heinrich Fritsch, M.D., Prof. Gynæcology and Obstetrics at the University of Hale. Translated by Isidor Furst. With 159 wood engravings. March, 1883. No. of Wood's Library.

The chapters of this work relating to the following subjects, are, alone, a most valuable contribution to this interesting and rapidly developing department of practical medicine: Diseases of the Pelvic Connective Tissue, the Pelvic Peritoneum, the Uterine Ligaments and the Tubes; Diseases of the Ovaries, and lastly, Hysteria.

